# Supplementary material for: Investigating the Antioxidant Efficiency of Tea Flavonoid Derivatives: A Density Functional Theory Study
Source: Int J Mol Sci. 2025 Mar 13;26(6):2587. doi: 10.3390/ijms26062587 (PMC11942223; doi:10.3390/ijms26062587)
Supplement: Supplementary file 1 [file ijms-26-02587-s001.zip › ijms-3485165-supplementary.pdf]

Supplementary Information for:

Investigating the Antioxidant Efficiency of Tea Flavonoid Derivatives: A Density Functional Theory Study

Yingmin Hou [1,†], Yuxi Wang [2,†], Xiaofei Tan [1], Yi Wang [1,*], Wenzhi Li [2,*] and Xianzhen Li [1]

[1] School of Biological Engineering, Dalian Polytechnic University, Dalian 116034, China; yingminhou@163.com (Y.H.); txf20232024@163.com (X.T.); xianzhen@dlpu.edu.cn (X.L.)
[2] Institute of Frontier Chemistry, School of Chemistry and Chemical Engineering, Shandong University, Qingdao 266237, China; yx.wang@mail.sdu.edu.cn
* Correspondence: wangyi@dlpu.edu.cn (Y.W.); 202217024@mail.sdu.edu.cn (W.L.)
† These authors contributed equally to this work.

Table S1. Calculated bond dissociation enthalpy (BDEs) of the studied compounds (kcal/mol).

	Compound	BDE (3-OH)	BDE (5-OH)	BDE (7-OH)	BDE (3'-OH)	BDE (4'-OH)	BDE (5'(2')-OH)
**Gas phase**							
**1**	Genkwanin	-	107.2	-	-	83.1	-
**2**	Apigenin	-	106.9	87.9	-	83.2	-
**3**	Luteolin	-	106.9	88.0	84.9	74.2	-
**4**	Acacetin	-	94.2	81.3	-	-	-
**5**	Wogonin	-	91.4	74.2	-	-	-
**6**	Chrysin	-	100.6	88.3	-	-	-
**7**	Kaempferol	72.4	99.1	87.7	-	82.8	-
**8**	Rhamnetin	60.8	75.9	-	57.4	54.6	-
**9**	Fisetin	72.9	-	85.3	76.0	74.0	-
**10**	Quercetin	70.5	85.7	85.8	75.5	82.3	-
**11**	Myricetin	74.7	74.0	87.9	74.8	65.9	74.0
**12**	Morin	75.7	106.8	87.4	-	83.4	83.0
**13**	Afzelechin	95.0	77.6	78.0	-	77.8	-
**14**	Catechin	99.3	80.4	81.7	74.5	82.5	-
**15**	Epicatechin	96.9	82.0	81.7	79.5	79.4	-
**16**	Gallocatechin	99.9	80.4	83.0	77.0	75.6	83.9
**17**	Liquiritigenin	-	-	86.2	-	83.1	-
**18**	Naringenin	-	84.9	103.3	-	82.9	-
**19**	Hesperitin	-	86.0	80.9	71.3	-	-
**20**	Eriodictyol	-	85.1	86.5	75.0	82.9	-
**21**	Pinocembrin	-	85.0	103.4	-	-	-
**22**	Aromadendrin	97.1	89.7	82.0	-	75.5	-
**23**	Taxifolin	104.8	97.5	103.6	83.8	74.4	-
**24**	Dihydromyricetin	104.6	97.6	90.1	83.8	75.8	76.2
**25**	Dihydromorin	104.5	97.4	89.5	-	82.5	81.5
**Solvent=Water**							
**1**	Genkwanin	-	104.8	-	-	113.3	-
**2**	Apigenin	-	104.6	87.3	-	112.1	-
**3**	Luteolin	-	104.6	87.2	81.4	107.2	-
**4**	Acacetin	-	94.5	117.5	-	-	-
**5**	Wogonin	-	89.4	109.8	-	-	-
**6**	Chrysin	-	94.5	114.4	-	-	-
**7**	Kaempferol	103.9	92.5	86.3	-	114.4	-
**8**	Rhamnetin	76.4	88.8	-	76.5	109.3	-
**9**	Fisetin	103.7	-	85.6	76.9	75.2	-
**10**	Quercetin	105.1	83.1	86.0	76.5	79.2	-
**11**	Myricetin	74.1	92.6	86.8	76.6	106.2	75.9
**12**	Morin	109.2	92.4	86.5	-	83.9	83.3
**13**	Afzelechin	99.6	107.9	80.8	-	82.2	-
**14**	Catechin	99.8	80.4	80.7	104.8	79.1	-
**15**	Epicatechin	100.0	80.8	80.5	78.3	107.1	-
**16**	Gallocatechin	99.9	79.8	81.3	108.1	72.8	80.0

**17**	Liquiritigenin	-	-	86.7	-	107.5	-
**18**	Naringenin	-	84.2	87.7	-	110.3	-
**19**	Hesperitin	-	84.3	87.8	109.4	-	-
**20**	Eriodictyol	-	84.2	87.7	106.0	79.2	-
**21**	Pinocembrin	-	109.4	87.8	-	-	-
**22**	Aromadendrin	103.3	92.2	89.8	-	113.2	-
**23**	Taxifolin	103.2	92.2	102.2	80.0	108.4	-
**24**	Dihydromyricetin	138.5	92.3	89.8	80.5	109.3	76.4
**25**	Dihydromorin	103.2	92.2	89.7	-	82.8	114.5
**Solvent=Ethanol**							
**1**	Genkwanin	-	125.3	-	-	113.3	-
**2**	Apigenin	-	75.8	58.4	-	83.3	-
**3**	Luteolin	-	105.2	87.5	81.8	75.8	-
**4**	Acacetin	-	94.8	117.6	-	-	-
**5**	Wogonin	-	120.3	109.8	-	-	-
**6**	Chrysin	-	94.8	114.4	-	-	-
**7**	Kaempferol	103.9	124.6	118.4	-	114.4	-
**8**	Rhamnetin	111.8	125.2	-	112.1	109.3	-
**9**	Fisetin	103.8	-	117.1	108.5	106.9	-
**10**	Quercetin	105.1	117.4	120.3	110.9	113.8	-
**11**	Myricetin	111.1	129.7	124.0	113.7	106.0	112.9
**12**	Morin	109.3	127.0	121.1	-	118.4	117.8
**13**	Afzelechin	99.5	108.0	80.9	-	82.2	-
**14**	Catechin	99.8	80.4	80.8	104.8	79.2	-
**15**	Epicatechin	99.8	80.9	80.5	107.7	107.3	-
**16**	Gallocatechin	99.9	79.8	113.2	108.2	105.2	80.2
**17**	Liquiritigenin	-	-	111.7	-	107.6	-
**18**	Naringenin	-	84.2	87.6	-	82.6	-
**19**	Hesperitin	-	115.9	119.3	109.4	-	-
**20**	Eriodictyol	-	84.2	87.6	106.0	79.4	-
**21**	Pinocembrin	-	109.5	113.0	-	-	-
**22**	Aromadendrin	103.4	92.5	89.8	-	113.3	-
**23**	Taxifolin	136.1	125.1	135.3	113.0	108.4	-
**24**	Dihydromyricetin	103.4	92.6	89.9	80.6	109.5	112.0
**25**	Dihydromorin	103.3	92.5	89.7	-	115.5	114.5

Table S2. Calculated proton dissociation enthalpy (PDEs) of the studied compounds (kcal/mol).

	Compound	PDE (3-OH)	PDE (5-OH)	PDE (7-OH)	PDE (3'-OH)	PDE (4'-OH)	PDE (5'(2')-OH)
**Gas phase**							
**1**	Genkwanin	-	280.1	-	-	256.0	-
**2**	Apigenin	-	277.9	259.0	-	254.3	-
**3**	Luteolin	-	282.6	263.7	260.5	249.9	-
**4**	Acacetin	-	275.1	262.1	-	-	-
**5**	Wogonin	-	277.7	260.6	-	-	-
**6**	Chrysin	-	264.6	252.3	-	-	-
**7**	Kaempferol	251.8	278.5	267.1	-	262.2	-
**8**	Rhamnetin	269.3	284.3	-	265.9	263.0	-
**9**	Fisetin	252.9	-	265.3	256.0	254.0	-
**10**	Quercetin	256.2	271.4	271.5	261.2	268.0	-
**11**	Myricetin	264.4	263.6	277.5	264.4	255.5	263.6
**12**	Morin	259.9	291.0	271.7	-	267.6	267.3
**13**	Afzelechin	274.4	256.9	257.4	-	257.1	-
**14**	Catechin	279.3	260.4	261.8	254.6	262.6	-
**15**	Epicatechin	275.6	260.7	260.5	258.3	258.1	-
**16**	Gallocatechin	280.6	261.1	263.7	257.7	256.4	264.6
**17**	Liquiritigenin	-	-	248.8	-	245.7	-
**18**	Naringenin	-	254.4	272.7	-	252.3	-
**19**	Hesperitin	-	271.6	266.5	256.9	-	-
**20**	Eriodictyol	-	260.7	262.1	250.6	258.5	-
**21**	Pinocembrin	-	245.4	263.8	-	-	-
**22**	Aromadendrin	272.4	265.1	257.4	-	250.9	-
**23**	Taxifolin	279.6	272.3	278.4	258.7	249.2	-
**24**	Dihydromyricetin	283.8	276.8	269.3	263.1	255.1	255.4
**25**	Dihydromorin	278.4	271.4	263.5	-	256.5	255.5
**Solvent=Water**							
**1**	Genkwanin	-	281.0	-	-	289.5	-
**2**	Apigenin	-	281.1	263.8	-	288.6	-
**3**	Luteolin	-	283.6	266.2	260.4	286.2	-
**4**	Acacetin	-	271.7	294.8	-	-	-
**5**	Wogonin	-	275.0	295.3	-	-	-
**6**	Chrysin	-	268.5	288.4	-	-	-
**7**	Kaempferol	286.0	274.6	268.4	-	296.5	-
**8**	Rhamnetin	263.3	275.7	-	263.3	296.1	-
**9**	Fisetin	286.9	-	268.9	260.1	258.5	-
**10**	Quercetin	290.1	268.0	270.9	261.4	264.1	-
**11**	Myricetin	259.5	278.0	272.2	262.0	291.6	261.3
**12**	Morin	291.6	274.8	268.8	-	266.3	265.7
**13**	Afzelechin	281.4	289.7	262.6	-	264.0	-
**14**	Catechin	281.2	261.8	262.1	286.1	260.4	-
**15**	Epicatechin	282.6	263.5	263.1	261.0	289.7	-
**16**	Gallocatechin	281.7	261.6	263.1	289.9	254.6	261.8

**17**	Liquiritigenin	-	-	260.5	-	281.2	-
**18**	Naringenin	-	258.6	262.1	-	284.7	-
**19**	Hesperitin	-	266.6	270.0	291.7	-	-
**20**	Eriodictyol	-	264.7	268.2	286.6	259.8	-
**21**	Pinocembrin	-	282.4	260.7	-	-	-
**22**	Aromadendrin	275.7	264.7	262.2	-	285.7	-
**23**	Taxifolin	282.3	271.2	281.2	259.1	287.4	-
**24**	Dihydromyricetin	318.4	272.2	269.8	260.4	289.2	256.4
**25**	Dihydromorin	279.8	268.8	266.4	-	259.5	291.2
**Solvent=Ethanol**							
**1**	Genkwanin	-	300.5	-	-	288.5	-
**2**	Apigenin	-	280.3	262.9	-	287.7	-
**3**	Luteolin	-	314.5	296.8	291.1	285.1	-
**4**	Acacetin	-	271.2	294.0	-	-	-
**5**	Wogonin	-	304.9	294.4	-	-	-
**6**	Chrysin	-	267.8	287.4	-	-	-
**7**	Kaempferol	285.0	305.7	299.5	-	295.5	-
**8**	Rhamnetin	297.8	311.2	-	298.1	295.2	-
**9**	Fisetin	286.0	-	299.4	290.7	289.2	-
**10**	Quercetin	289.1	301.4	304.3	294.9	297.8	-
**11**	Myricetin	295.7	314.3	308.5	298.2	290.6	297.5
**12**	Morin	290.7	308.4	302.5	-	299.8	299.2
**13**	Afzelechin	280.3	288.8	261.7	-	262.9	-
**14**	Catechin	280.0	260.6	261.0	285.0	259.4	-
**15**	Epicatechin	281.5	262.6	262.2	289.4	289.0	-
**16**	Gallocatechin	280.7	260.6	294.1	289.0	286.0	261.0
**17**	Liquiritigenin	-	-	284.2	-	280.1	-
**18**	Naringenin	-	285.4	288.8	-	283.7	-
**19**	Hesperitin	-	297.1	300.6	290.7	-	-
**20**	Eriodictyol	-	263.7	267.1	285.4	258.8	-
**21**	Pinocembrin	-	281.3	284.8	-	-	-
**22**	Aromadendrin	274.7	263.8	261.1	-	284.6	-
**23**	Taxifolin	314.0	303.0	313.2	290.9	286.3	-
**24**	Dihydromyricetin	282.2	271.4	268.7	259.5	288.3	290.8
**25**	Dihydromorin	278.8	268.0	265.3	-	291.0	290.1

Table S3. Calculated proton affinity (PAs) of the studied compounds (kcal/mol).

	Compound	PA (3-OH)	PA (5-OH)	PA (7-OH)	PA (3'-OH)	PA (4'-OH)	PA (5'(2')-OH)
**Gas phase**							
**1**	Genkwanin	-	350.1	-	-	321.6	-
**2**	Apigenin	-	347.8	329.2	-	321.6	-
**3**	Luteolin	-	348.2	329.7	335.7	314.1	-
**4**	Acacetin	-	348.3	329.6	-	-	-
**5**	Wogonin	-	346.8	324.3	-	-	-
**6**	Chrysin	-	346.7	328.2	-	-	-
**7**	Kaempferol	325.1	347.5	329.2	-	322.5	-
**8**	Rhamnetin	332.5	339.3	-	326.9	319.4	-
**9**	Fisetin	329.2	-	328.0	321.0	318.1	-
**10**	Quercetin	327.9	333.5	328.5	321.5	331.1	-
**11**	Myricetin	324.7	347.3	329.0	314.8	307.0	314.3
**12**	Morin	331.7	350.2	331.7	-	322.2	322.5
**13**	Afzelechin	357.2	338.6	341.9	-	334.1	-
**14**	Catechin	357.3	335.2	339.1	326.9	339.2	-
**15**	Epicatechin	360.6	341.5	340.1	337.4	338.3	-
**16**	Gallocatechin	355.7	332.2	336.5	327.5	332.7	340.6
**17**	Liquiritigenin	-	-	328.4	-	336.2	-
**18**	Naringenin	-	331.4	327.6	-	337.3	-
**19**	Hesperitin	-	330.1	326.2	335.7	-	-
**20**	Eriodictyol	-	330.4	326.5	330.0	341.9	-
**21**	Pinocembrin	-	331.1	327.4	-	-	-
**22**	Aromadendrin	352.5	338.6	322.3	-	335.4	-
**23**	Taxifolin	353.2	339.2	323.1	343.6	326.8	-
**24**	Dihydromyricetin	350.6	338.2	322.4	343.1	332.4	328.7
**25**	Dihydromorin	322.5	340.9	324.6	-	332.6	330.7
**Solvent=Water**							
**1**	Genkwanin	-	328.2	-	-	315.1	-
**2**	Apigenin	-	325.7	314.2	-	313.8	-
**3**	Luteolin	-	328.2	316.6	322.9	311.4	-
**4**	Acacetin	-	327.2	315.6	-	-	-
**5**	Wogonin	-	326.9	313.6	-	-	-
**6**	Chrysin	-	322.7	311.3	-	-	-
**7**	Kaempferol	316.2	327.7	316.7	-	317.8	-
**8**	Rhamnetin	324.3	328.3	-	321.0	317.2	-
**9**	Fisetin	317.7	-	316.8	314.9	313.4	-
**10**	Quercetin	320.2	320.7	319.6	317.7	322.8	-
**11**	Myricetin	320.6	332.5	321.6	318.0	313.1	304.4
**12**	Morin	322.7	330.3	319.3	-	320.7	319.8
**13**	Afzelechin	334.6	318.7	320.1	-	316.8	-
**14**	Catechin	337.1	320.6	322.2	314.4	320.9	-
**15**	Epicatechin	339.6	321.6	322.8	320.7	321.1	-
**16**	Gallocatechin	339.1	321.7	323.9	318.4	320.2	324.6

**17**	Liquiritigenin	-	-	309.9	-	316.3	-
**18**	Naringenin	-	313.7	312.7	-	319.4	-
**19**	Hesperitin	-	317.2	316.2	322.2	-	-
**20**	Eriodictyol	-	316.1	315.0	316.8	323.4	-
**21**	Pinocembrin	-	311.0	310.0	-	-	-
**22**	Aromadendrin	334.2	321.7	312.4	-	321.0	-
**23**	Taxifolin	336.6	324.1	314.8	326.3	318.0	-
**24**	Dihydromyricetin	339.0	326.7	317.4	328.0	322.4	321.4
**25**	Dihydromorin	316.1	324.5	315.1	-	322.5	321.2
**Solvent=Ethanol**							
**1**	Genkwanin	-	330.1	-	-	316.2	-
**2**	Apigenin	-	298.6	286.7	-	286.1	-
**3**	Luteolin	-	298.6	286.7	293.0	281.0	-
**4**	Acacetin	-	329.0	317.0	-	-	-
**5**	Wogonin	-	328.6	314.9	-	-	-
**6**	Chrysin	-	324.4	312.6	-	-	-
**7**	Kaempferol	317.6	329.5	318.1	-	318.9	-
**8**	Rhamnetin	325.8	329.9	-	322.3	318.3	-
**9**	Fisetin	319.2	-	318.1	316.1	314.5	-
**10**	Quercetin	321.6	322.3	320.9	318.8	324.2	-
**11**	Myricetin	322.1	334.4	323.0	319.0	314.0	305.6
**12**	Morin	324.3	332.2	320.8	-	321.8	321.0
**13**	Afzelechin	336.4	320.3	321.7	-	345.9	-
**14**	Catechin	339.0	322.1	323.7	315.7	322.6	-
**15**	Epicatechin	341.6	323.3	324.3	322.2	322.7	-
**16**	Gallocatechin	340.9	323.0	325.3	319.7	321.6	326.2
**17**	Liquiritigenin	-	-	311.3	-	317.8	-
**18**	Naringenin	-	287.4	286.2	-	293.1	-
**19**	Hesperitin	-	318.7	317.5	323.8	-	-
**20**	Eriodictyol	-	317.5	316.3	318.2	325.1	-
**21**	Pinocembrin	-	312.5	311.3	-	-	-
**22**	Aromadendrin	336.1	323.3	313.6	-	322.5	-
**23**	Taxifolin	338.5	325.7	316.1	328.0	319.4	-
**24**	Dihydromyricetin	341.0	328.3	318.7	329.9	324.1	322.8
**25**	Dihydromorin	317.3	326.2	316.4	-	324.0	322.6

Table S4. Calculated electron transfer enthalpy (ETEs) of the studied compounds (kcal/mol)

	Compound	ETE (3-OH)	ETE (5-OH)	ETE (7-OH)	ETE (3'-OH)	ETE (4'-OH)	ETE (5'(2')-OH)
**Gas phase**							
**1**	Genkwanin	-	68.6	-	-	73.0	-
**2**	Apigenin	-	70.6	70.3	-	73.2	-
**3**	Luteolin	-	70.2	69.8	60.7	71.7	-
**4**	Acacetin	-	63.9	69.7	-	-	-
**5**	Wogonin	-	61.2	66.6	-	-	-
**6**	Chrysin	-	65.4	71.6	-	-	-
**7**	Kaempferol	58.8	63.2	70.1	-	71.8	-
**8**	Rhamnetin	58.9	67.1	-	61.1	65.7	-
**9**	Fisetin	55.2	-	68.8	66.6	67.4	-
**10**	Quercetin	54.1	63.7	68.8	65.6	62.7	-
**11**	Myricetin	61.5	38.2	70.3	71.5	70.4	71.3
**12**	Morin	55.5	68.2	67.2	-	72.7	72.1
**13**	Afzelechin	53.8	54.9	52.0	-	59.6	-
**14**	Catechin	53.5	56.7	54.2	59.2	54.9	-
**15**	Epicatechin	47.8	52.0	53.2	53.6	52.6	-
**16**	Gallocatechin	55.7	59.8	58.1	61.0	54.4	54.9
**17**	Liquiritigenin	-	-	69.3	-	58.4	-
**18**	Naringenin	-	65.1	87.2	-	57.1	-
**19**	Hesperitin	-	73.1	71.9	52.8	-	-
**20**	Eriodictyol	-	66.2	71.5	56.6	52.5	-
**21**	Pinocembrin	-	65.4	87.6	-	-	-
**22**	Aromadendrin	63.9	70.5	79.0	-	59.4	-
**23**	Taxifolin	63.1	69.8	92.0	51.8	59.1	-
**24**	Dihydromyricetin	65.5	70.9	79.3	52.3	55.0	59.0
**25**	Dihydromorin	93.6	68.0	76.5	-	61.4	62.3
**Solvent=Water**							
**1**	Genkwanin	-	88.2	-	-	109.7	-
**2**	Apigenin	-	90.5	84.6	-	109.9	-
**3**	Luteolin	-	88.0	82.2	70.1	107.3	-
**4**	Acacetin	-	78.8	113.5	-	-	-
**5**	Wogonin	-	74.1	107.7	-	-	-
**6**	Chrysin	-	83.3	114.6	-	-	-
**7**	Kaempferol	99.2	76.3	81.2	-	108.1	-
**8**	Rhamnetin	63.7	72.1	-	67.0	103.6	-
**9**	Fisetin	97.5	-	80.4	73.4	73.3	-
**10**	Quercetin	96.5	73.9	77.9	70.3	67.9	-
**11**	Myricetin	65.1	71.6	76.8	70.1	104.6	74.0
**12**	Morin	98.0	73.7	78.7	-	74.8	75.0
**13**	Afzelechin	76.5	100.7	72.3	-	76.9	-
**14**	Catechin	74.2	71.3	70.0	101.9	69.6	-
**15**	Epicatechin	71.9	70.7	69.2	69.2	97.5	-
**16**	Gallocatechin	72.3	69.6	69.0	101.2	64.2	66.9

**17**	Liquiritigenin	-	-	88.3	-	102.7	-
**18**	Naringenin	-	82.0	86.5	-	102.5	-
**19**	Hesperitin	-	78.6	83.1	98.7	-	-
**20**	Eriodictyol	-	79.6	84.2	100.7	67.4	-
**21**	Pinocembrin	-	110.0	89.3	-	-	-
**22**	Aromadendrin	80.6	82.0	89.0	-	103.7	-
**23**	Taxifolin	78.2	79.7	98.9	65.3	101.9	-
**24**	Dihydromyricetin	111.0	77.1	84.0	64.0	98.4	66.6
**25**	Dihydromorin	98.6	79.2	86.1	-	71.8	104.8
**Solvent=Ethanol**							
**1**	Genkwanin	-	88.2	-	-	109.7	-
**2**	Apigenin	-	90.5	84.6	-	109.9	-
**3**	Luteolin	-	88.0	82.2	70.1	107.3	-
**4**	Acacetin	-	78.8	113.5	-	-	-
**5**	Wogonin	-	74.1	107.7	-	-	-
**6**	Chrysin	-	83.3	114.6	-	-	-
**7**	Kaempferol	99.2	76.3	81.2	-	108.1	-
**8**	Rhamnetin	63.7	72.1	-	67.0	103.6	-
**9**	Fisetin	97.5	-	80.4	73.4	73.3	-
**10**	Quercetin	96.5	73.9	77.9	70.3	67.9	-
**11**	Myricetin	65.1	71.6	76.8	70.1	104.6	74.0
**12**	Morin	98.0	73.7	78.7	-	74.8	75.0
**13**	Afzelechin	76.5	100.7	72.3	-	76.9	-
**14**	Catechin	74.2	71.3	70.0	101.9	69.6	-
**15**	Epicatechin	71.9	70.7	69.2	69.2	97.5	-
**16**	Gallocatechin	72.3	69.6	69.0	101.2	64.2	66.9
**17**	Liquiritigenin	-	-	88.3	-	102.7	-
**18**	Naringenin	-	82.0	86.5	-	102.5	-
**19**	Hesperitin	-	78.6	83.1	98.7	-	-
**20**	Eriodictyol	-	79.6	84.2	100.7	67.4	-
**21**	Pinocembrin	-	110.0	89.3	-	-	-
**22**	Aromadendrin	80.6	82.0	89.0	-	103.7	-
**23**	Taxifolin	78.2	79.7	98.9	65.3	101.9	-
**24**	Dihydromyricetin	111.0	77.1	84.0	64.0	98.4	66.6
**25**	Dihydromorin	98.6	79.2	86.1	-	71.8	104.8

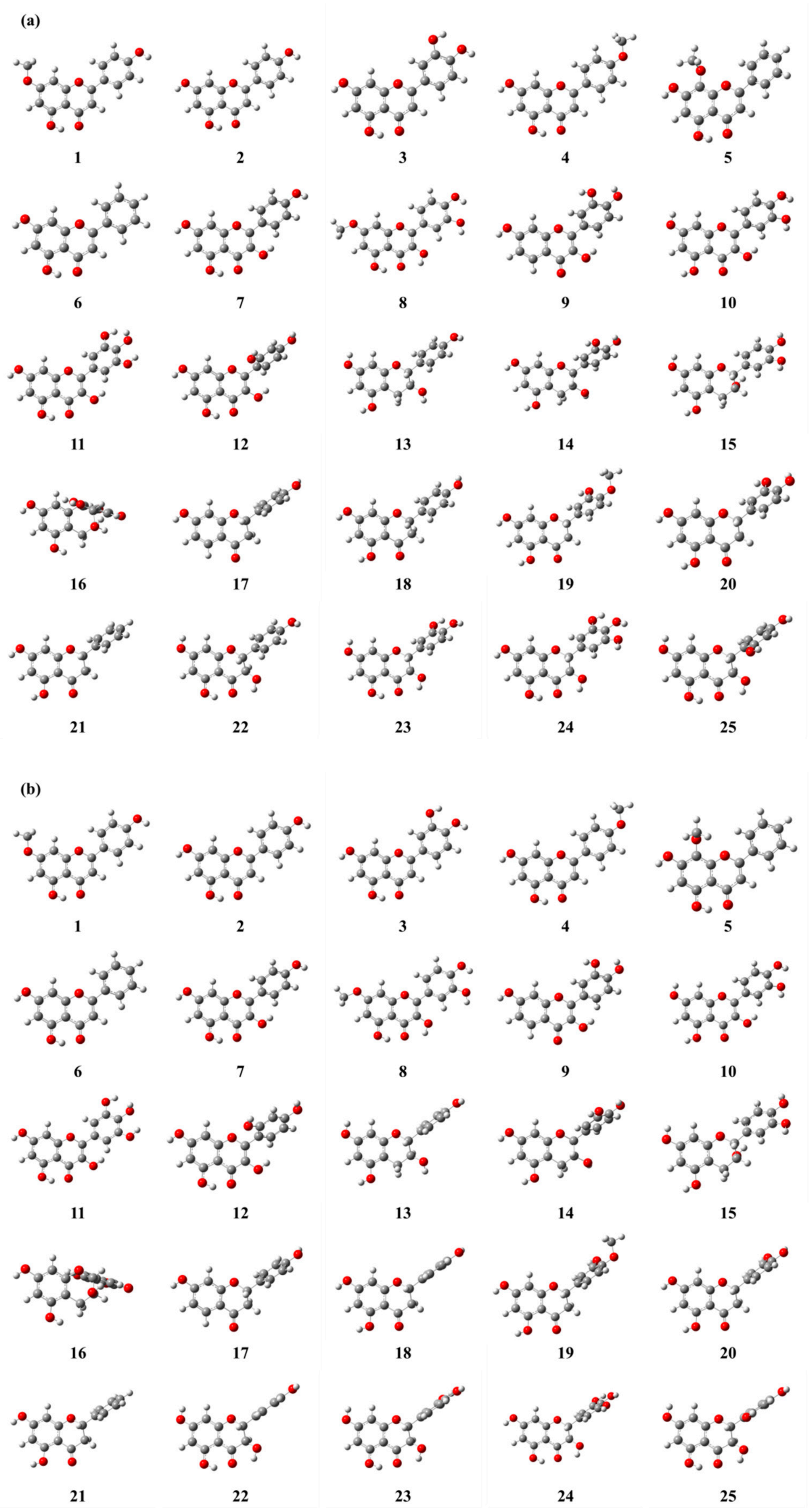
(a)
1
2
3
4
5
6
7
8
9
10
11
12
13
14
15
16
17
18
19
20
21
22
23
24
25
(b)
1
2
3
4
5
6
7
8
9
10
11
12
13
14
15
16
17
18
19
20
21
22
23
24
25

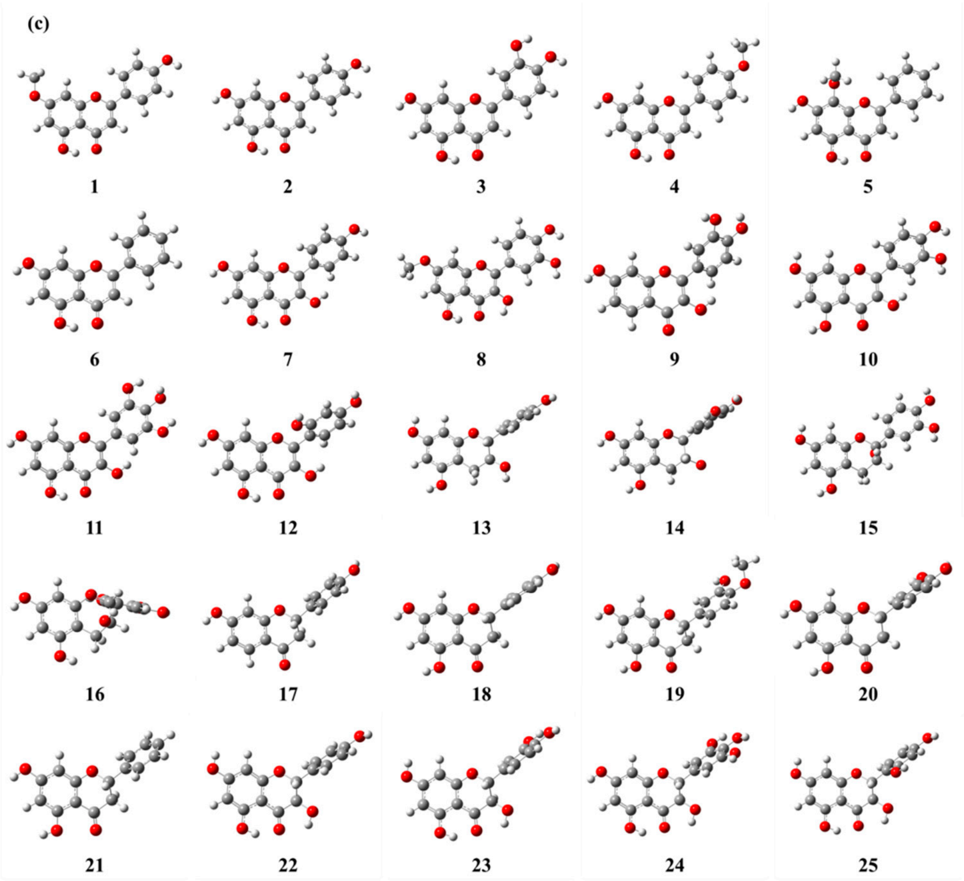

Figure S1. The optimized geometrical structures (a) Gas phase (b) Water medium (c) Ethanol medium.

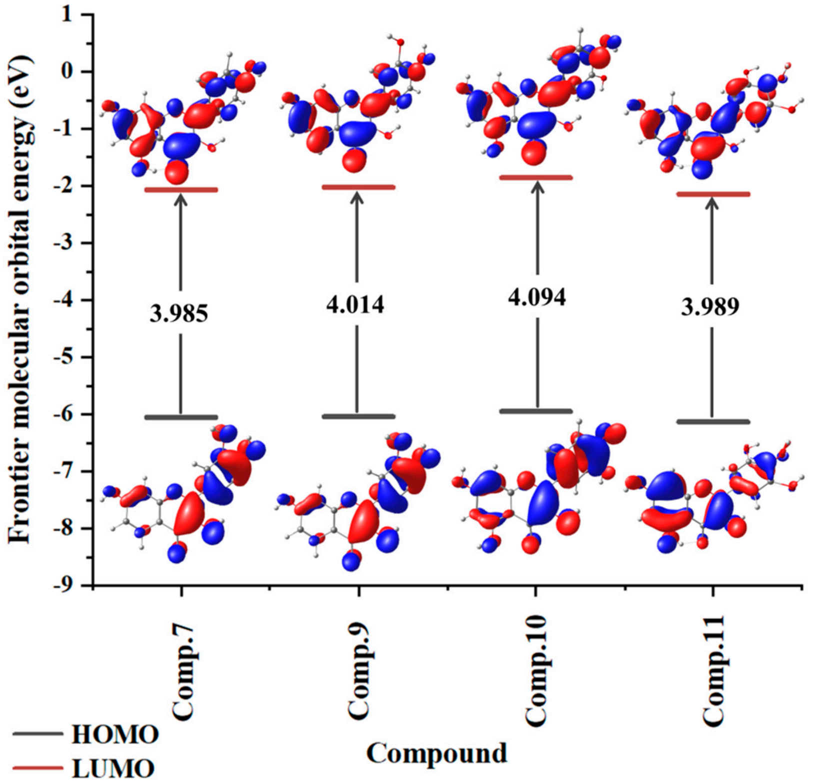

Figure S2. Neutral E_{HOMO}, E_{LUMO} and the $E_{LUMO-HOMO}$ of compound 7, 9-11 in water medium.

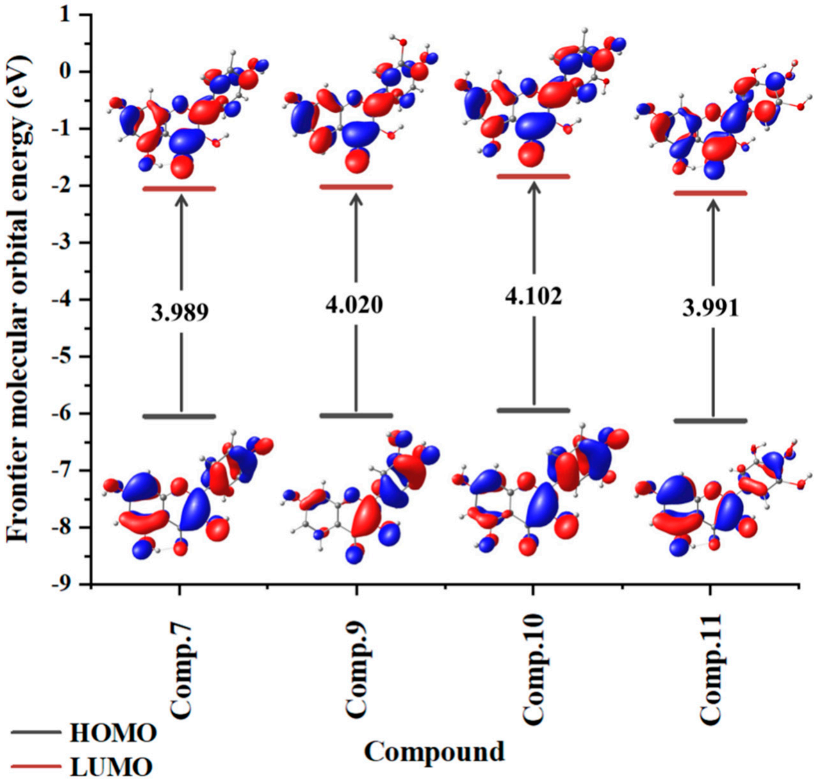

Figure S3. Neutral E_{HOMO}, E_{LUMO} and the $E_{LUMO-HOMO}$ of compound 7, 9-11 in ethanol medium.

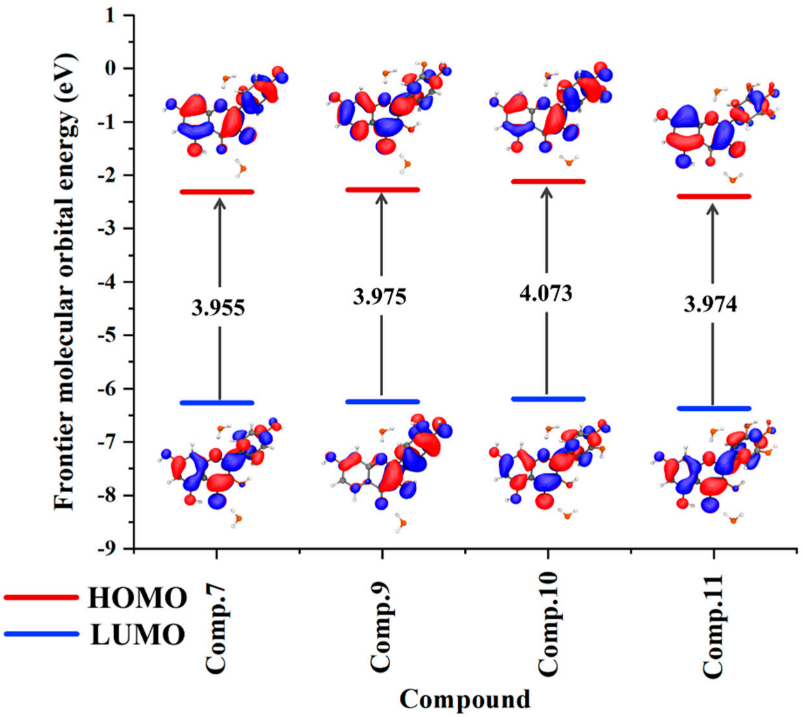

Figure S4. Complex EHOMO, ELUMO and the ELUMO-HOMO of compound 7, 9-11 in ethanol medium.

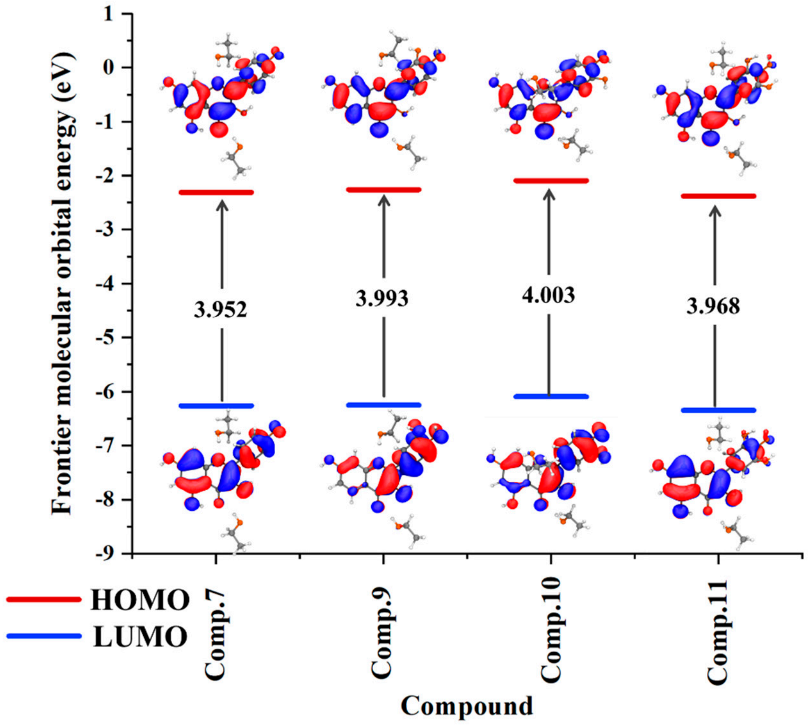

Figure S5. Complex EHOMO, ELUMO and the ELUMO-HOMO of compound 7, 9-11 in ethanol medium.

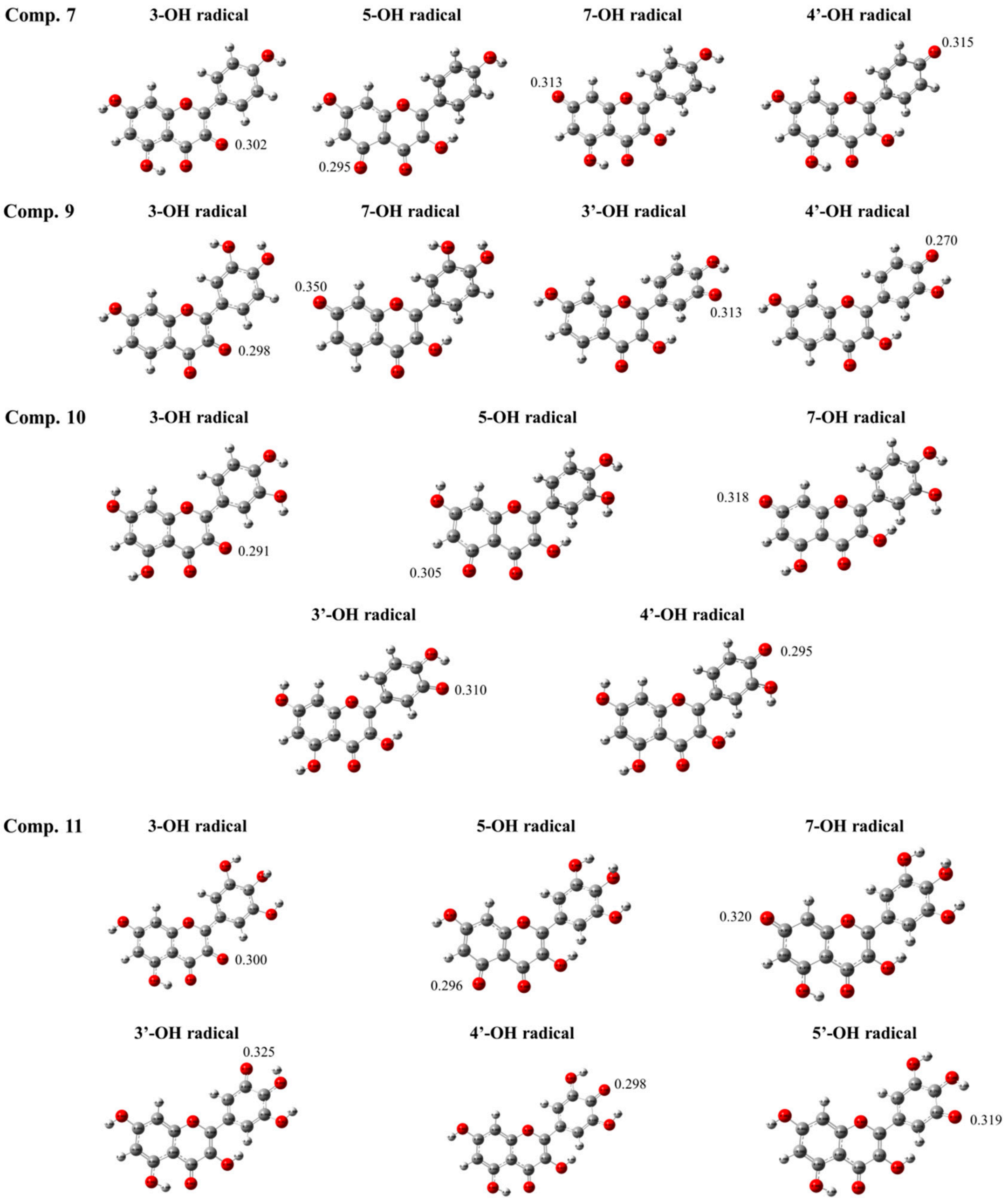

Figure S6. Spin density distribution of structural radicals 7, 9-11 obtained after H-atom abstraction in water medium.

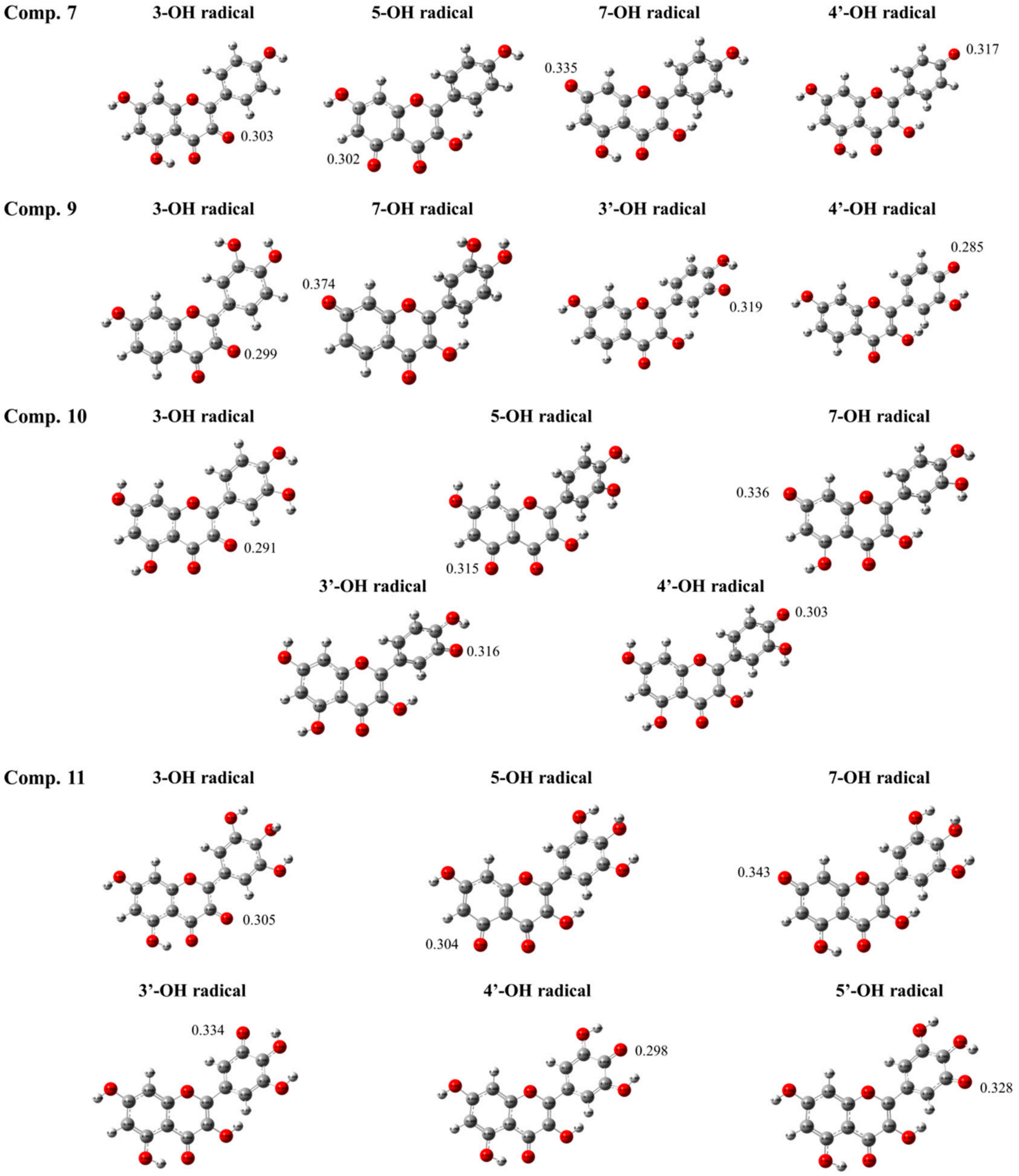

Figure S7. Spin density distribution of structural radicals 7, 9-11 obtained after H-atom abstraction in ethanol medium.

B3LYP-D3(BJ)/TZVP optimized geometries in gas phase

**Kaempferol-OPT**			
**Atom**	**x**	**y**	**z**
O	-0.106395	-0.835005	0.068705
O	0.867405	2.665915	-0.275003
O	-4.156122	1.714971	-0.075291
O	-1.833238	2.849133	-0.177677
O	-4.23876	-3.03537	0.199299
O	6.149818	-0.995475	-0.017764
C	-2.090006	0.508106	-0.002207
C	-1.465061	-0.744749	0.060145
C	0.67116	0.288447	0.00064
C	-1.312932	1.723438	-0.100776
C	0.132989	1.530446	-0.120764
C	2.104053	-0.023478	0.012324
C	-3.510777	0.546489	-0.00579
C	-2.174002	-1.931694	0.130804
C	-3.562989	-1.856718	0.129953
C	-4.235124	-0.63388	0.064741
C	3.010735	0.750153	0.745075
C	2.592526	-1.127718	-0.700321
C	4.364858	0.446543	0.748863
C	3.93938	-1.436869	-0.699127
C	4.834295	-0.645295	0.021756
H	1.777846	2.432748	-0.499107
H	-3.45927	2.424595	-0.127685
H	-5.18822	-2.867432	0.196171
H	6.671111	-0.386617	0.518151
H	-1.667642	-2.883404	0.18613
H	-5.316973	-0.585327	0.06335
H	2.65402	1.572917	1.351591
H	1.901175	-1.745037	-1.257503
H	5.05226	1.050026	1.331183
H	4.319414	-2.285137	-1.252317

Zero-point correction= 0.223983 (Hartree/Particle)
Thermal correction to Energy= 0.241099
Thermal correction to Enthalpy= 0.242044
Thermal correction to Gibbs Free Energy= 0.179161
Sum of electronic and zero-point Energies= -1029.164235
Sum of electronic and thermal Energies= -1029.147119
Sum of electronic and thermal Enthalpies= -1029.146174
Sum of electronic and thermal Free Energies= -1029.209057

**Kaempferol-O3·**			
**Atom**	**x**	**y**	**z**
O	-0.068119	-0.749786	0.011865
O	0.808917	2.714033	-0.014856
O	-4.214299	1.651175	-0.008797
O	-1.932447	2.87167	0.009797
O	-4.105642	-3.10202	0.012499
O	6.147477	-1.035297	-0.005246
C	-2.104188	0.528375	-0.018939
C	-1.431697	-0.699941	0.004766
C	0.719752	0.355912	0.006843
C	-1.375084	1.769154	0.001463
C	0.135406	1.677502	-0.003839
C	2.122978	0.040817	0.002434
C	-3.525828	0.510025	-0.008103
C	-2.091293	-1.911381	0.003979
C	-3.488317	-1.893763	0.005344
C	-4.207734	-0.700742	-0.006912
C	3.122693	1.037899	0.021144
C	2.541373	-1.311288	-0.017235
C	4.460434	0.695888	0.018409
C	3.875389	-1.64949	-0.019524
C	4.847647	-0.645207	-0.001684
H	-3.548649	2.391649	-0.003547
H	-5.062912	-2.98351	0.009284
H	6.725382	-0.262852	0.007713
H	-1.550577	-2.845488	0.01755
H	-5.290437	-0.693094	-0.003548
H	2.82849	2.074459	0.035635
H	1.798995	-2.094621	-0.03122
H	5.212998	1.47674	0.032797
H	4.190437	-2.684035	-0.035646

Zero-point correction= 0.210834 (Hartree/Particle)
Thermal correction to Energy= 0.227878
Thermal correction to Enthalpy= 0.228823
Thermal correction to Gibbs Free Energy= 0.164715
Sum of electronic and zero-point Energies= -1028.548884
Sum of electronic and thermal Energies= -1028.531840
Sum of electronic and thermal Enthalpies= -1028.530895
Sum of electronic and thermal Free Energies= -1028.595003

**Kaempferol-TS**			
**Atom**	**x**	**y**	**z**
O	0.254132	-1.068101	-0.129299
O	-0.783842	2.253163	-0.923413
O	4.270063	1.529655	-0.409957
O	1.932216	2.575512	-0.818231
O	4.392362	-3.11617	0.591034
O	-5.941096	-1.626086	-0.028508
C	2.221651	0.306535	-0.274405
C	1.612553	-0.936193	-0.063554
C	-0.581289	-0.03801	-0.401158
C	1.436541	1.464691	-0.604275
C	-0.059238	1.27733	-0.66791
C	-1.969807	-0.40714	-0.328298
C	3.63943	0.375008	-0.201698
C	2.327295	-2.077329	0.231493
C	3.71983	-1.974318	0.302693
C	4.377951	-0.76474	0.094454
C	-3.012346	0.497067	-0.62641
C	-2.327822	-1.719483	0.062476
C	-4.333758	0.10804	-0.529388
C	-3.645273	-2.105931	0.156296
C	-4.661371	-1.192082	-0.139548
H	-1.143871	3.192936	0.628603
H	3.573409	2.212533	-0.605498
H	5.340564	-2.942042	0.624996
H	-6.554585	-0.916984	-0.256232
H	1.834619	-3.023729	0.39483
H	5.456687	-0.690828	0.150104
H	-2.767415	1.500765	-0.930954
H	-1.551301	-2.431885	0.295833
H	-5.119448	0.818249	-0.762466
H	-3.91411	-3.10908	0.458187
H	-1.796766	1.590692	2.28678
O	-1.402454	3.294793	1.568436
O	-0.949362	2.022072	2.10634

Zero-point correction= 0.239175 (Hartree/Particle)
Thermal correction to Energy= 0.259769
Thermal correction to Enthalpy= 0.260713
Thermal correction to Gibbs Free Energy= 0.187474
Sum of electronic and zero-point Energies= -1180.147752
Sum of electronic and thermal Energies= -1180.127157
Sum of electronic and thermal Enthalpies= -1180.126213
Sum of electronic and thermal Free Energies= -1180.199452

Kaempferol-In1			
**Atom**	**x**	**y**	**z**
O	-0.546973	-0.998076	0.087594
O	0.695392	2.424622	0.302752
O	-4.400867	1.84063	0.147311
O	-2.009703	2.800425	0.31718
O	-4.818431	-2.877148	-0.321438
O	5.683934	-1.554584	0.448566
C	-2.428568	0.485047	0.134627
C	-1.894184	-0.805702	0.028186
C	0.30314	0.063617	0.233634
C	-1.566215	1.642214	0.2461
C	-0.13452	1.350623	0.252713
C	1.706087	-0.356389	0.311422
C	-3.840799	0.629905	0.066901
C	-2.683645	-1.933443	-0.1196
C	-4.062014	-1.755304	-0.175064
C	-4.645745	-0.490023	-0.080654
C	2.571862	0.216875	1.248232
C	2.20096	-1.35279	-0.540169
C	3.899307	-0.168979	1.315519
C	3.529086	-1.738185	-0.486214
C	4.385273	-1.143294	0.44322
H	1.488621	2.259188	-0.237444
H	-3.653723	2.494891	0.235293
H	-5.752527	-2.639135	-0.342551
H	6.172845	-1.096225	1.14216
H	-2.245966	-2.917748	-0.188882
H	-5.719988	-0.360538	-0.12598
H	2.201229	0.977613	1.921334
H	1.535521	-1.816486	-1.255441
H	4.559655	0.285191	2.045676
H	3.917003	-2.509018	-1.139097
H	4.189253	0.602068	-1.932308
O	2.618982	1.534346	-1.668446
O	3.936157	1.436683	-1.488003

Zero-point correction=	0.239932 (Hartree/Particle)
Thermal correction to Energy=	0.261055
Thermal correction to Enthalpy=	0.261999
Thermal correction to Gibbs Free Energy=	0.188902
Sum of electronic and zero-point Energies=	-1180.129308
Sum of electronic and thermal Energies=	-1180.108185
Sum of electronic and thermal Enthalpies=	-1180.107241
Sum of electronic and thermal Free Energies=	-1180.180338

Kaempferol-In2			
Atom	x	y	z
O	0.661063	-1.101564	0.034359
O	-0.864186	2.124387	-0.004901
O	4.249874	2.058265	0.092952
O	1.779564	2.807826	0.154554
O	5.075487	-2.614281	-0.123051
O	-5.407184	-2.482229	0.030641
C	2.406738	0.545861	0.05668
C	1.986857	-0.787945	0.007785
C	-0.320106	-0.172204	0.073808
C	1.451487	1.618232	0.103148
C	-0.00406	1.231873	0.05767
C	-1.637282	-0.74206	0.096543
C	3.803844	0.806562	0.046573
C	2.868953	-1.845479	-0.051825
C	4.235377	-1.554656	-0.063425
C	4.708107	-0.245702	-0.014516
C	-2.769698	0.010855	0.464957
C	-1.82797	-2.097708	-0.255796
C	-4.026938	-0.551929	0.45188
C	-3.08258	-2.65973	-0.270577
C	-4.194762	-1.884529	0.07507
H	3.446807	2.648196	0.130301
H	5.990825	-2.310035	-0.125366
H	-6.09596	-1.847813	0.263987
H	2.521023	-2.866508	-0.083605
H	5.768472	-0.028607	-0.023251
H	-2.66413	1.033766	0.778602
H	-0.97577	-2.697312	-0.538006
H	-4.881497	0.055176	0.725798
H	-3.23154	-3.691436	-0.557607
H	-2.446834	2.64104	-0.764958
H	-3.994578	3.860617	0.47487
O	-3.341864	2.930503	-1.038464
O	-4.030832	2.923876	0.235333

Zero-point correction=	0.241835 (Hartree/Particle)
Thermal correction to Energy=	0.262650
Thermal correction to Enthalpy=	0.263595
Thermal correction to Gibbs Free Energy=	0.189576
Sum of electronic and zero-point Energies=	-1180.204964
Sum of electronic and thermal Energies=	-1180.184149
Sum of electronic and thermal Enthalpies=	-1180.183204
Sum of electronic and thermal Free Energies=	-1180.257223

**Fisetin-OPT**			
**Atom**	**x**	**y**	**z**
O	0.558501	-0.587028	-0.174667
O	-0.367754	2.899952	0.392631
O	2.335164	3.041848	0.412324
O	4.64461	-2.874621	-0.3359
O	-4.094587	-2.270545	0.971067
O	-5.706502	-0.577659	-0.326925
C	-0.200921	0.550701	-0.052293
C	2.571488	0.705476	0.072854
C	1.91509	-0.514226	-0.110523
C	-1.637313	0.26663	-0.134441
C	0.354614	1.768019	0.181456
C	1.812103	1.951276	0.234375
C	3.971915	0.697841	0.120266
C	2.608661	-1.712346	-0.248171
C	-2.150792	-0.887798	0.473711
C	-2.513864	1.114537	-0.819418
C	3.991902	-1.686695	-0.198077
C	4.680169	-0.475291	-0.012603
C	-3.500432	-1.168546	0.405337
C	-3.873047	0.830623	-0.879798
C	-4.378355	-0.304503	-0.265503
H	-1.299125	2.671787	0.51426
H	5.596808	-2.730651	-0.292094
H	-3.437839	-2.826813	1.403408
H	-5.872596	-1.405367	0.145274
H	4.474326	1.644811	0.266205
H	2.078119	-2.641747	-0.397374
H	-1.481086	-1.561516	0.9943
H	-2.13049	1.974129	-1.353126
H	5.763748	-0.470907	0.024591
H	-4.554629	1.476502	-1.416719

Zero-point correction= 0.223602 (Hartree/Particle)
Thermal correction to Energy= 0.241123
Thermal correction to Enthalpy= 0.242068
Thermal correction to Gibbs Free Energy= 0.178307
Sum of electronic and zero-point Energies= -1029.151223
Sum of electronic and thermal Energies= -1029.133702
Sum of electronic and thermal Enthalpies= -1029.132758
Sum of electronic and thermal Free Energies= -1029.196518

Fisetin-O3·			
**Atom**	**x**	**y**	**z**
O	-0.068119	-0.749786	0.011865
O	0.808917	2.714033	-0.014856
O	-4.214299	1.651175	-0.008797
O	-1.932447	2.87167	0.009797
O	-4.105642	-3.10202	0.012499
O	6.147477	-1.035297	-0.005246
C	-2.104188	0.528375	-0.018939
C	-1.431697	-0.699941	0.004766
C	0.719752	0.355912	0.006843
C	-1.375084	1.769154	0.001463
C	0.135406	1.677502	-0.003839
C	2.122978	0.040817	0.002434
C	-3.525828	0.510025	-0.008103
C	-2.091293	-1.911381	0.003979
C	-3.488317	-1.893763	0.005344
C	-4.207734	-0.700742	-0.006912
C	3.122693	1.037899	0.021144
C	2.541373	-1.311288	-0.017235
C	4.460434	0.695888	0.018409
C	3.875389	-1.64949	-0.019524
C	4.847647	-0.645207	-0.001684
H	-3.548649	2.391649	-0.003547
H	-5.062912	-2.98351	0.009284
H	6.725382	-0.262852	0.007713
H	-1.550577	-2.845488	0.01755
H	-5.290437	-0.693094	-0.003548
H	2.82849	2.074459	0.035635
H	1.798995	-2.094621	-0.03122
H	5.212998	1.47674	0.032797
H	4.190437	-2.684035	-0.035646

Zero-point correction= 0.209490 (Hartree/Particle)
Thermal correction to Energy= 0.227313
Thermal correction to Enthalpy= 0.228257
Thermal correction to Gibbs Free Energy= 0.162197
Sum of electronic and zero-point Energies= -1028.535413
Sum of electronic and thermal Energies= -1028.517591
Sum of electronic and thermal Enthalpies= -1028.516646
Sum of electronic and thermal Free Energies= -1028.582706

**Fisetin-TS**			
**Atom**	**x**	**y**	**z**
O	0.474555	-0.910151	-0.13036
O	-0.283625	2.511626	-0.882088
O	2.444492	2.567586	-0.817269
O	4.404331	-3.382934	0.444136
O	-3.99058	-2.867821	0.34949
O	-5.746709	-0.875947	0.047087
C	-0.254178	0.220806	-0.337815
C	2.566805	0.258574	-0.314573
C	1.835324	-0.907217	-0.101688
C	-1.681615	-0.031798	-0.259532
C	0.389696	1.427947	-0.599197
C	1.87229	1.514778	-0.595001
C	3.962784	0.178633	-0.267414
C	2.447299	-2.127667	0.15128
C	-2.130582	-1.343928	0.000953
C	-2.647773	0.97858	-0.414432
C	3.83182	-2.177039	0.19331
C	4.595229	-1.018466	-0.016342
C	-3.473442	-1.621237	0.100435
C	-3.996717	0.6904	-0.307889
C	-4.42726	-0.603594	-0.0518
H	-0.040739	3.30865	-0.155541
H	5.36438	-3.29455	0.448735
H	-3.282523	-3.515981	0.426188
H	-5.855153	-1.821794	0.219621
H	4.52454	1.087388	-0.435084
H	1.859757	-3.019597	0.312824
H	-1.413884	-2.143379	0.124624
H	-2.331662	1.985474	-0.620718
H	5.676953	-1.072873	0.020082
H	-4.738394	1.468281	-0.427286
H	-0.063955	2.806481	2.491693
O	-0.029578	3.921193	1.000755
O	0.597439	2.983855	1.802385

Zero-point correction= 0.236310 (Hartree/Particle)
Thermal correction to Energy= 0.256740
Thermal correction to Enthalpy= 0.257685
Thermal correction to Gibbs Free Energy= 0.184018
Sum of electronic and zero-point Energies= -1180.171537
Sum of electronic and thermal Energies= -1180.151107
Sum of electronic and thermal Enthalpies= -1180.150163
Sum of electronic and thermal Free Energies= -1180.223829

Fisetin-In1			
**Atom**	**x**	**y**	**z**
O	0.973913	-0.755643	-0.17971
O	-0.260143	2.675757	-0.138044
O	2.420224	3.052035	-0.067327
O	5.230621	-2.680047	0.135072
O	-3.592894	-2.669758	0.932915
O	-5.236631	-1.348229	-0.719014
C	0.119418	0.315785	-0.255565
C	2.858447	0.724564	-0.057426
C	2.311755	-0.55926	-0.0814
C	-1.275458	-0.115415	-0.392173
C	0.556316	1.599436	-0.163787
C	1.993532	1.90827	-0.098192
C	4.250641	0.84436	0.0364
C	3.10522	-1.700372	-0.016628
C	-1.737756	-1.211293	0.342889
C	-2.160637	0.539629	-1.252412
C	4.477204	-1.548816	0.07427
C	5.055909	-0.269326	0.101144
C	-3.053507	-1.618362	0.236285
C	-3.479494	0.119176	-1.368369
C	-3.940095	-0.95116	-0.619908
H	-1.144392	2.447779	0.199334
H	6.163988	-2.445378	0.188549
H	-2.91303	-3.109791	1.454455
H	-5.356087	-2.142697	-0.181019
H	4.667216	1.842142	0.058622
H	2.658466	-2.683627	-0.044432
H	-1.062292	-1.73596	1.007115
H	-1.813019	1.372143	-1.846574
H	6.132625	-0.16742	0.172732
H	-4.16463	0.618168	-2.039833
H	-4.173478	1.195908	1.6548
O	-2.985469	2.326924	0.817838
O	-3.235163	1.44214	1.776453

Zero-point correction= 0.240751 (Hartree/Particle)
Thermal correction to Energy= 0.262064
Thermal correction to Enthalpy= 0.263008
Thermal correction to Gibbs Free Energy= 0.189077
Sum of electronic and zero-point Energies= -1180.172340
Sum of electronic and thermal Energies= -1180.151027
Sum of electronic and thermal Enthalpies= -1180.150083
Sum of electronic and thermal Free Energies= -1180.224014

**Fisetin-In2**			
**Atom**	**x**	**y**	**z**
O	-0.880053	-0.907751	-0.011998
O	0.493763	2.317179	-0.627922
O	-2.161263	2.94331	-0.322916
O	-5.210321	-2.627707	0.34255
O	3.194724	-3.689651	-0.075928
O	5.247916	-1.99934	0.101747
C	0.059303	0.058575	-0.166897
C	-2.713776	0.656391	-0.093603
C	-2.217257	-0.64277	0.00829
C	1.401334	-0.444153	-0.112361
C	-0.329652	1.430106	-0.366954
C	-1.799681	1.785096	-0.25754
C	-4.102027	0.834796	-0.042358
C	-3.050917	-1.741552	0.15503
C	1.617369	-1.843053	-0.123671
C	2.524057	0.406199	-0.014711
C	-4.421692	-1.533858	0.199654
C	-4.951746	-0.238313	0.100867
C	2.888704	-2.352885	-0.05793
C	3.798147	-0.120638	0.060573
C	4.00025	-1.494742	0.034248
H	-6.137705	-2.364444	0.365791
H	2.391979	-4.218885	-0.130205
H	5.19485	-2.965559	0.078099
H	-4.481946	1.844271	-0.121135
H	-2.643152	-2.738306	0.237996
H	0.774516	-2.516504	-0.194566
H	2.394787	1.474557	0.019356
H	-6.024575	-0.090704	0.137593
H	4.656842	0.530166	0.150327
H	1.121379	3.809108	0.168239
H	3.348172	4.413627	-0.288598
O	1.710206	4.423821	0.653478
O	3.003823	3.823574	0.39615

Zero-point correction= 0.240734 (Hartree/Particle)
Thermal correction to Energy= 0.262271
Thermal correction to Enthalpy= 0.263216
Thermal correction to Gibbs Free Energy= 0.186356
Sum of electronic and zero-point Energies= -1180.189657
Sum of electronic and thermal Energies= -1180.168120
Sum of electronic and thermal Enthalpies= -1180.167176
Sum of electronic and thermal Free Energies= -1180.244035

**Qurectin-OPT**			
**Atom**	**x**	**y**	**z**
C	-3.877336	-1.792945	0.244492
C	-4.521069	-0.557641	0.169699
C	-3.783377	0.607788	0.03807
C	-2.364932	0.572082	-0.021112
C	-1.770463	-0.695917	0.057339
C	-2.496824	-1.876075	0.190806
C	-1.523463	1.770197	-0.186668
C	-0.078001	1.474892	-0.263203
C	0.411158	0.222821	-0.115484
O	-0.422014	-0.858125	0.021742
C	1.826981	-0.160888	-0.136444
C	2.244861	-1.304278	-0.8216
C	3.584045	-1.667065	-0.843066
C	4.531097	-0.896152	-0.183585
C	4.117967	0.244456	0.516328
C	2.784265	0.609063	0.542945
O	-1.933925	2.914796	-0.269591
O	0.706004	2.565351	-0.489934
O	-4.673386	-2.88966	0.374394
O	-4.396868	1.809209	-0.036622
O	5.122388	0.92794	1.158675
O	5.839952	-1.256813	-0.212001
H	-5.602604	-0.527385	0.216633
H	-1.970375	-2.819359	0.252656
H	1.514311	-1.910089	-1.338726
H	3.916419	-2.548113	-1.375643
H	2.475819	1.465891	1.131474
H	1.585717	2.272367	-0.761603
H	-4.130037	-3.684821	0.418923
H	-5.351673	1.678436	0.009265
H	4.768917	1.686393	1.635802
H	6.346842	-0.616936	0.30682

Zero-point correction= 0.227426 (Hartree/Particle)
Thermal correction to Energy= 0.246325
Thermal correction to Enthalpy= 0.247270
Thermal correction to Gibbs Free Energy= 0.180402
Sum of electronic and zero-point Energies= -1104.391590
Sum of electronic and thermal Energies= -1104.372691
Sum of electronic and thermal Enthalpies= -1104.371747
Sum of electronic and thermal Free Energies= -1104.438614

**Qurectin-O3·**			
**Atom**	**x**	**y**	**z**
C	-3.84554	-1.804337	-0.004081
C	-4.517356	-0.584436	-0.019701
C	-3.802794	0.604842	-0.014601
C	-2.383022	0.605095	-0.000845
C	-1.760975	-0.649315	0.014443
C	-2.458043	-1.849896	0.008862
C	-1.563033	1.822455	0.026664
C	-0.045454	1.608768	-0.012771
C	0.459084	0.260177	0.007573
O	-0.404764	-0.788698	0.030346
C	1.836978	-0.145313	0.002954
C	2.172897	-1.5177	0.004164
C	3.491048	-1.928761	0.001789
C	4.518122	-0.99021	-0.001908
C	4.196819	0.377121	-0.003382
C	2.889114	0.799525	0.000675
O	-1.994324	2.954634	0.070979
O	0.694181	2.597306	-0.051512
O	-4.611892	-2.926143	-0.00734
O	-4.438349	1.793625	-0.029604
O	5.28175	1.219795	-0.007107
O	5.807777	-1.395834	-0.004968
H	-5.600147	-0.58205	-0.031975
H	-1.912158	-2.783833	0.020554
H	1.386829	-2.256462	0.006991
H	3.750146	-2.978963	0.002094
H	2.647954	1.852566	-0.002523
H	-4.05083	-3.710283	0.004573
H	-5.391851	1.645638	-0.044894
H	4.992468	2.139168	-0.012245
H	6.375491	-0.611351	-0.00793

Zero-point correction= 0.213750 (Hartree/Particle)

Thermal correction to Energy= 0.232736

Thermal correction to Enthalpy= 0.233681

Thermal correction to Gibbs Free Energy= 0.164951

Sum of electronic and zero-point Energies= -1103.779360

Sum of electronic and thermal Energies= -1103.760374

Sum of electronic and thermal Enthalpies= -1103.759430

Sum of electronic and thermal Free Energies= -1103.828159

Qurectin-TS			
Atom	x	y	z
C	3.605424	-2.585772	-0.071544
C	4.348112	-1.401516	-0.04365
C	3.709158	-0.176125	-0.01956
C	2.287749	-0.098679	-0.009142
C	1.590962	-1.317027	-0.041061
C	2.222185	-2.557435	-0.065172
C	1.531403	1.138342	-0.010477
C	0.077045	1.00704	0.015613
C	-0.524983	-0.219272	-0.011437
O	0.242749	-1.352643	-0.043838
C	-1.953082	-0.530279	0.024676
C	-2.367269	-1.839508	0.321989
C	-3.713847	-2.159654	0.368118
C	-4.670359	-1.187722	0.11875
C	-4.269789	0.106618	-0.224943
C	-2.926817	0.439737	-0.254033
O	2.053376	2.265168	-0.014218
O	-0.668296	2.124194	0.052482
O	4.315588	-3.743428	-0.099166
O	4.412625	0.976887	0.005162
O	-5.20037	1.075063	-0.506279
O	-6.012695	-1.51662	0.142922
H	5.429117	-1.46431	-0.048319
H	1.623862	-3.458346	-0.089009
H	-1.63187	-2.602151	0.52584
H	-4.042979	-3.159702	0.617337
H	-2.649875	1.449603	-0.506702
H	3.716509	-4.498906	-0.117855
H	5.355733	0.772387	-0.000276
H	-5.979655	0.652009	-0.89015
H	-6.481549	-0.898279	0.718481
H	1.969081	3.858919	0.073836
O	0.339533	4.696148	0.197854
O	1.659448	4.811947	0.143431
H	-0.11941	2.942568	0.101261

Zero-point correction= 0.243186 (Hartree/Particle)
Thermal correction to Energy= 0.264957
Thermal correction to Enthalpy= 0.265901
Thermal correction to Gibbs Free Energy= 0.189799
Sum of electronic and zero-point Energies= -1255.380004
Sum of electronic and thermal Energies= -1255.358233
Sum of electronic and thermal Enthalpies= -1255.357288
Sum of electronic and thermal Free Energies= -1255.433391

Qurectin-In1			
**Atom**	**x**	**y**	**z**
C	-4.209477	-1.796572	0.457475
C	-4.735904	-0.526989	0.216163
C	-3.895379	0.531087	-0.086943
C	-2.48906	0.34843	-0.154004
C	-2.013086	-0.949194	0.092967
C	-2.843947	-2.021309	0.40016
C	-1.53943	1.420061	-0.47929
C	-0.152902	0.954378	-0.605693
C	0.238528	-0.291028	-0.261052
O	-0.683646	-1.243932	0.072916
C	1.620521	-0.775394	-0.218556
C	1.955728	-2.048722	-0.683055
C	3.272417	-2.486549	-0.645214
C	4.273609	-1.662844	-0.14795
C	3.939667	-0.388376	0.330431
C	2.629614	0.049961	0.304206
O	-1.807804	2.602929	-0.633844
O	0.711876	1.92092	-1.052613
O	-5.101652	-2.780149	0.749573
O	-4.387384	1.764082	-0.332623
O	4.986614	0.344929	0.82638
O	5.558157	-2.0975	-0.119418
H	-5.808471	-0.387119	0.267745
H	-2.408768	-2.993442	0.590158
H	1.183666	-2.694559	-1.077132
H	3.545502	-3.468773	-1.007051
H	2.39068	1.019277	0.728661
H	1.592825	1.54081	-1.176791
H	-4.637538	-3.611274	0.903078
H	-5.349681	1.741706	-0.266265
H	4.687263	1.216514	1.111647
H	6.110025	-1.40057	0.262672
H	0.731658	3.420908	-0.061527
O	2.267408	3.393833	0.969501
O	1.163611	4.029248	0.595996

Zero-point correction= 0.244481 (Hartree/Particle)
Thermal correction to Energy= 0.266966
Thermal correction to Enthalpy= 0.267911
Thermal correction to Gibbs Free Energy= 0.190593
Sum of electronic and zero-point Energies= -1255.367715
Sum of electronic and thermal Energies= -1255.345230
Sum of electronic and thermal Enthalpies= -1255.344285
Sum of electronic and thermal Free Energies= -1255.421603

Qurectin-In2			
**Atom**	**x**	**y**	**z**
C	4.499644	-1.520633	-0.036203
C	4.962401	-0.208282	0.008165
C	4.063422	0.846874	0.031524
C	2.663333	0.614725	0.011979
C	2.25521	-0.722871	-0.031464
C	3.138831	-1.792084	-0.056675
C	1.656203	1.677597	0.036139
C	0.19951	1.219623	0.035937
C	-0.079523	-0.189444	-0.030748
O	0.94272	-1.078038	-0.060296
C	-1.370118	-0.812363	-0.038867
C	-1.50393	-2.209897	0.120044
C	-2.751845	-2.797918	0.154598
C	-3.90146	-2.018258	0.041395
C	-3.782479	-0.628009	-0.12698
C	-2.54181	-0.045006	-0.186907
O	1.894205	2.865995	0.061828
O	-0.683336	2.08686	0.106044
O	5.435976	-2.500716	-0.058102
O	4.495864	2.120418	0.074711
O	-4.95001	0.064277	-0.229533
O	-5.119682	-2.591865	0.095181
H	6.029626	-0.027637	0.02342
H	2.754356	-2.80223	-0.093817
H	-0.623239	-2.822849	0.231467
H	-2.862999	-3.8657	0.285347
H	-2.463948	1.009736	-0.353566
H	5.009195	-3.364756	-0.090176
H	5.460836	2.130185	0.084902
H	-4.773403	1.019411	-0.129413
H	-5.783006	-1.889262	0.016677
H	-2.254327	3.023876	-0.154826
H	-4.418666	3.440346	0.89881
O	-3.131062	3.315439	-0.486306
O	-4.02452	2.675699	0.457668

Zero-point correction= 0.245965 (Hartree/Particle)
Thermal correction to Energy= 0.268115
Thermal correction to Enthalpy= 0.269059
Thermal correction to Gibbs Free Energy= 0.192348
Sum of electronic and zero-point Energies= -1255.446837
Sum of electronic and thermal Energies= -1255.424687
Sum of electronic and thermal Enthalpies= -1255.423743
Sum of electronic and thermal Free Energies= -1255.500454

Myricetin-OPT			
**Atom**	**x**	**y**	**z**
O	0.56623	-0.769174	0.179888
O	-0.323436	2.697568	-0.538382
O	4.672438	1.652243	-0.263379
O	2.37376	2.821942	-0.482596
O	4.646733	-3.03525	0.555062
O	-4.728649	1.557668	1.239763
O	-3.934931	-2.470584	-1.204435
O	-5.715165	-0.736949	0.03508
C	2.579043	0.509847	-0.051279
C	-0.185703	0.3567	-0.012179
C	1.925583	-0.712219	0.159384
C	-1.627767	0.074368	0.01559
C	0.376824	1.567494	-0.265292
C	1.830191	1.724322	-0.278287
C	4.000623	0.514788	-0.05992
C	2.607472	-1.899397	0.364467
C	-2.104664	-1.085691	-0.599063
C	-2.510093	0.967788	0.635122
C	3.997683	-1.857275	0.352115
C	4.697685	-0.66622	0.143655
C	-3.872968	0.69283	0.636539
C	-3.466683	-1.35219	-0.592108
C	-4.342608	-0.483603	0.058662
H	-1.258445	2.481732	-0.657263
H	3.992828	2.367923	-0.39382
H	5.599768	-2.891372	0.527994
H	-5.632719	1.284318	1.028728
H	-4.899269	-2.407907	-1.254201
H	-5.955806	-1.282907	0.796116
H	2.079376	-2.826204	0.529739
H	-1.43216	-1.771222	-1.09282
H	-2.157487	1.840179	1.167863
H	5.78034	-0.643505	0.135213

Zero-point correction= 0.232556 (Hartree/Particle)
Thermal correction to Energy= 0.252021
Thermal correction to Enthalpy= 0.252965
Thermal correction to Gibbs Free Energy= 0.185165
Sum of electronic and zero-point Energies= -1179.659440
Sum of electronic and thermal Energies= -1179.639975
Sum of electronic and thermal Enthalpies= -1179.639031
Sum of electronic and thermal Free Energies= -1179.706831

**Myricetin-O4′·**			
**Atom**	**x**	**y**	**z**
O	-0.525654	0.768703	0.156644
O	0.359076	-2.707754	-0.498397
O	-4.627584	-1.677011	-0.213869
O	-2.317536	-2.846451	-0.398003
O	-4.60972	3.031561	0.453448
O	4.828756	-1.601694	1.018813
O	3.932296	2.632859	-0.9246
O	5.652422	0.762425	0.003076
C	-2.536352	-0.523573	-0.037965
C	0.240262	-0.353995	0.002435
C	-1.885508	0.707044	0.13312
C	1.664069	-0.064738	0.029775
C	-0.327504	-1.577714	-0.230146
C	-1.78533	-1.738848	-0.229296
C	-3.958725	-0.533015	-0.047295
C	-2.569677	1.896196	0.300391
C	2.109777	1.186755	-0.448838
C	2.577228	-1.024417	0.532379
C	-3.961686	1.850116	0.288675
C	-4.658903	0.651768	0.118435
C	3.921951	-0.747292	0.529573
C	3.451217	1.471513	-0.462322
C	4.425762	0.509929	0.013163
H	1.290589	-2.505272	-0.662425
H	-3.951322	-2.397407	-0.320841
H	-5.563464	2.890014	0.431436
H	5.699166	-1.174022	0.912529
H	4.902231	2.589078	-0.833684
H	-2.045593	2.829955	0.436586
H	1.400177	1.911839	-0.817607
H	2.23364	-1.929713	1.013896
H	-5.741364	0.626047	0.110711
Zero-point correction=		0.220905 (Hartree/Particle)	
Thermal correction to Energy=		0.239692	
Thermal correction to Enthalpy=		0.240637	
Thermal correction to Gibbs Free Energy=		0.173475	
Sum of electronic and zero-point Energies=		-1179.053810	
Sum of electronic and thermal Energies=		-1179.035023	
Sum of electronic and thermal Enthalpies=		-1179.034079	
Sum of electronic and thermal Free Energies=		-1179.101240	

Myricetin-TS			
**Atom**	**x**	**y**	**z**
O	-1.014012	0.683759	-0.054988
O	-0.457413	-2.908929	-0.354988
O	-5.312076	-1.415773	0.174015
O	-3.130111	-2.797038	-0.055392
O	-4.865581	3.318215	0.306139
O	4.17916	-1.972237	0.793272
O	3.448962	1.923987	-1.845672
O	5.168106	0.098007	-0.704067
C	-3.129153	-0.43882	0.055896
C	-0.362907	-0.514675	-0.142515
C	-2.371152	0.740744	0.036866
C	1.086573	-0.352643	-0.266252
C	-1.036912	-1.698559	-0.1813
C	-2.495803	-1.730567	-0.05432
C	-4.542944	-0.323559	0.158643
C	-2.942014	1.997447	0.122994
C	1.587006	0.732396	-0.997111
C	1.956501	-1.277387	0.337591
C	-4.328342	2.073138	0.224796
C	-5.130053	0.929291	0.244853
C	3.323801	-1.115642	0.192799
C	2.954058	0.891701	-1.135993
C	3.839951	-0.031182	-0.54319
H	0.472123	-2.800126	-0.599129
H	-4.704661	-2.199431	0.095622
H	-5.825746	3.257829	0.372651
H	5.078086	-1.748547	0.50419
H	4.414617	1.837497	-1.871969
H	5.582516	0.645424	0.09731
H	-2.335294	2.890151	0.111154
H	0.918299	1.443177	-1.458522
H	1.59164	-2.066174	0.980773
H	-6.207785	0.998302	0.324126
H	4.295316	0.958323	2.470007
O	5.781546	1.226334	1.368828
O	4.485071	1.530361	1.704258

Zero-point correction= 0.245126 (Hartree/Particle)
Thermal correction to Energy= 0.267574
Thermal correction to Enthalpy= 0.268518
Thermal correction to Gibbs Free Energy= 0.191193
Sum of electronic and zero-point Energies= -1330.627204
Sum of electronic and thermal Energies= -1330.604755
Sum of electronic and thermal Enthalpies= -1330.603811
Sum of electronic and thermal Free Energies= -1330.681136

Myricetin-In1			
**Atom**	**x**	**y**	**z**
O	-1.140491	0.767389	0.007086
O	-0.36253	-2.703664	-0.810965
O	-5.279524	-1.638611	0.110481
O	-3.031147	-2.819618	-0.39974
O	-5.130561	3.05289	0.889229
O	4.257832	-1.571651	0.460998
O	3.178334	2.493524	-1.818835
O	5.05698	0.780446	-0.8609
C	-3.171571	-0.504802	0.045655
C	-0.424455	-0.362301	-0.27466
C	-2.49245	0.716177	0.161583
C	1.011438	-0.088256	-0.419481
C	-1.020692	-1.569959	-0.452544
C	-2.46315	-1.722772	-0.273199
C	-4.582596	-0.503327	0.219008
C	-3.137424	1.907119	0.445327
C	1.426711	1.080032	-1.064399
C	1.961794	-0.984958	0.086215
C	-4.518246	1.871135	0.612024
C	-5.243291	0.681882	0.503377
C	3.306758	-0.697718	-0.071546
C	2.778399	1.358309	-1.200583
C	3.732855	0.472238	-0.687024
H	0.532832	-2.48221	-1.099325
H	-4.626302	-2.358604	-0.102114
H	-6.079778	2.914377	0.987209
H	4.942773	-1.726944	-0.205321
H	4.14729	2.515238	-1.802768
H	5.525973	0.70896	0.000557
H	-2.589363	2.832682	0.535547
H	0.703707	1.782728	-1.452242
H	1.675652	-1.869471	0.638319
H	-6.318121	0.663854	0.634112
H	5.031461	-0.95071	1.862007
O	6.110891	0.552408	1.72102
O	5.598891	-0.406449	2.479815

Zero-point correction=	0.249902 (Hartree/Particle)
Thermal correction to Energy=	0.272542
Thermal correction to Enthalpy=	0.273486
Thermal correction to Gibbs Free Energy=	0.196657
Sum of electronic and zero-point Energies=	-1330.633808
Sum of electronic and thermal Energies=	-1330.611168
Sum of electronic and thermal Enthalpies=	-1330.610224
Sum of electronic and thermal Free Energies=	-1330.687053

Myricetin-In2			
**Atom**	**x**	**y**	**z**
O	-1.133939	0.637049	0.224464
O	-0.779067	-2.892675	-0.587145
O	-5.54084	-1.107056	-0.402955
O	-3.441284	-2.612668	-0.586428
O	-4.818865	3.49596	0.527231
O	3.747194	-2.586649	1.098297
O	3.523771	1.900862	-0.523902
O	4.975361	-0.370319	0.343106
C	-3.310117	-0.306833	-0.102994
C	-0.545904	-0.579503	0.034294
C	-2.481389	0.792547	0.158294
C	0.901499	-0.522475	0.13435
C	-1.289949	-1.685257	-0.280979
C	-2.752761	-1.612153	-0.338461
C	-4.714733	-0.091532	-0.152747
C	-2.977026	2.064937	0.369823
C	1.55861	0.670021	-0.215436
C	1.635462	-1.646759	0.586617
C	-4.357098	2.239947	0.316067
C	-5.226591	1.178201	0.058
C	3.001265	-1.571832	0.651911
C	2.932618	0.766792	-0.168254
C	3.722003	-0.371637	0.262598
H	0.177396	-2.832375	-0.716218
H	-4.974128	-1.915284	-0.53013
H	-5.781332	3.506722	0.467333
H	4.673661	-2.281913	1.05536
H	4.507317	1.873055	-0.460066
H	-2.319245	2.895833	0.573495
H	0.989741	1.52455	-0.548226
H	1.14303	-2.525886	0.977212
H	-6.298083	1.326765	0.01812
H	6.392026	0.46156	-0.30586
H	6.621897	2.748633	0.246051
O	7.030156	1.12448	-0.649238
O	6.22406	2.319959	-0.525376

Zero-point correction= 0.251402 (Hartree/Particle)
Thermal correction to Energy= 0.273723
Thermal correction to Enthalpy= 0.274667
Thermal correction to Gibbs Free Energy= 0.198051
Sum of electronic and zero-point Energies= -1330.723182
Sum of electronic and thermal Energies= -1330.700861
Sum of electronic and thermal Enthalpies= -1330.699917
Sum of electronic and thermal Free Energies= -1330.776533
